# Fetal mesenchymal hamartoma of the liver: A case report and literature review

**DOI:** 10.3389/fped.2022.1016260

**Published:** 2022-11-23

**Authors:** Xin Liu, Sha Tian, Zhenchuang Zhu, Fei Peng, Qiang Yuan, Xufei Duan, Xueqiang Yan

**Affiliations:** ^1^ Department of Pediatric Surgery, Wuhan Children's Hospital (Wuhan Maternal and Child Healthcare Hospital), Tongji Medical College, Huazhong University of Science & Technology, Wuhan, China; ^2^ Department of Ultrasound, Wuhan Children's Hospital (Wuhan Maternal and Child Healthcare Hospital), Tongji Medical College, Huazhong University of Science & Technology, Wuhan, China

**Keywords:** mesenchymal hamartoma, liver tumor, fetus, prenatal examination, treat

## Abstract

This paper reports the diagnosis and treatment process of a case of fetal mesenchymal hamartoma of the liver (MHL), and reviews the previous literature reports. At 38^+2^ weeks of gestation, prenatal ultrasound found a well bound mixed solid and cystic mass, which was located at the lower edge of the right lobe of the liver and in front of the right kidney of the fetus, but the source and nature of the mass were not clear by ultrasound. Due to the approaching due date, the fetus showed no other abnormal symptoms, and no special treatment was given with the consent of the family members. A female fetus was delivered weighing 3,520 g at 39 weeks. An exploratory laparotomy was performed on the eighth day after delivery. During the operation, it was found that the tumor originated from the fifth, sixth and seventh hepatic segment and the corresponding hepatic segments were removed. Recovery was uneventful and the infant was discharged on the 6th day after surgery. Follow-up at 2 years showed a thriving young girl, and there was no tumor recurrence.

## Introduction

MHL is the second most common benign liver tumor in children after infantile hemangioma, accounting for 6%–8% of liver tumors in children (1), and about 80% of them are found in infants under 2 years old (2). MHL can occur as early as the fetus, and there are often no obvious symptoms in the early stage. The tumor can grow rapidly in the abdominal cavity of the fetus, which may cause the death of the fetus in the intrauterine and neonatal period (3). At present, there are a few reports about fetal MHL, most of which are individual cases. The prenatal diagnosis rate is low, and there is no unified treatment plan. It is necessary to summarize the cases of fetal HML in order to understand and treat them comprehensively.

## Materials and methods

### Case report

**Figure 1:**
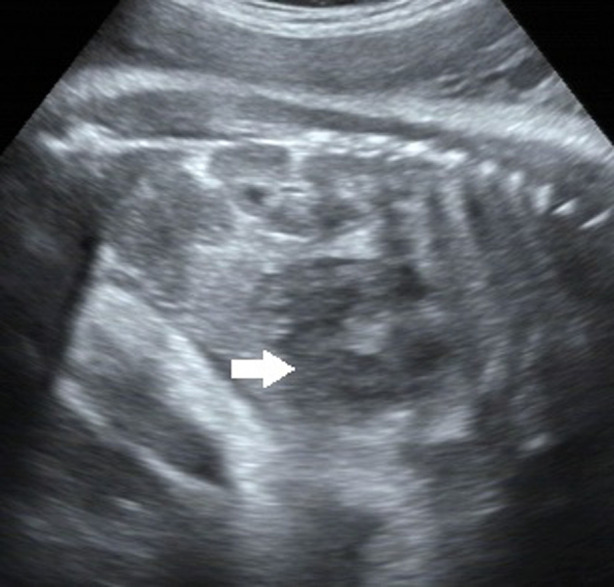
A well-defined 46 mm × 29 mm mixed mass at the lower margin of the right lobe and in front of the right kidney, which is polycystic with uneven internal echo. Arrows point to the mass.

A 38-year-old G3P2 female was admitted to our hospital for routine prenatal examination at 38^+2^ weeks of gestation. Past medical history was negative for medical illnesses, drug or medication exposure, smoking or alcohol ingestion. Diabetes screening was negative. Family history was likewise unremarkable. Maternal serum alpha-feto protein screening was not performed. Ultrasound examination at 34 weeks showed no significant abnormalities. The examination at 38 weeks, however, revealed an intraabdominal well-bounded mixed solid and cystic mass measuring 46 × 29 mm. The mass was located at the lower edge of the right lobe of the fetal liver and in front of the right kidney. It was polycystic with uneven internal echo (Figure 1). The Color Doppler Flow Imaging indicated that there was no obvious blood flow signal. Both kidneys, abdominal wall, stomach bubble, and bladder were normal. There was no evidence of fetal edema. Fetal development was consistent with fetal age. The amniotic fluid index was 7.2 cm. Amniocentesis was not performed. Due to the approaching due date, the fetus showed no other abnormal symptoms, and no special treatment was given with the consent of the family members.

**Figure 2:**
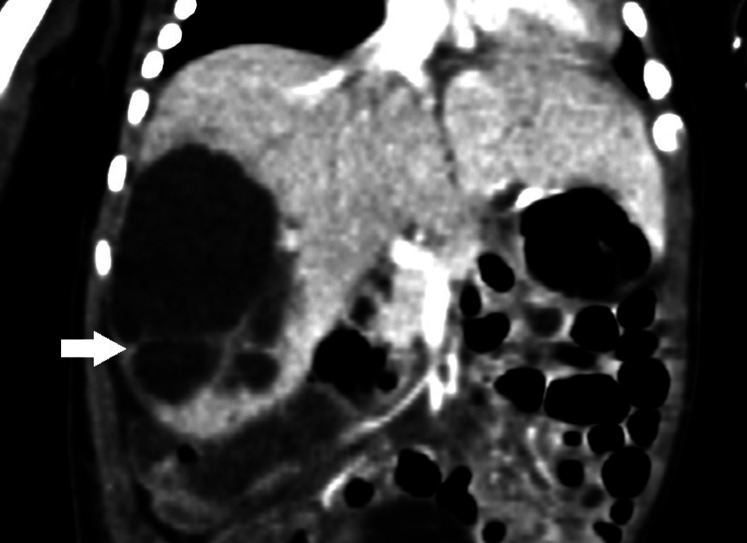
Computed tomography (CT) scans of the abdomen. CT scans showed a circular cystic mass in the right lobe of the liver, with multiple linear partitions and clear boundaries. On contrast enhanced CT scans, the capsule and septum enhanced, but no enhancement was found inside the tumor (arrow).

A female fetus weighing 3,520 g was delivered at 39 weeks. The newborn had no acute respiratory distress, the abdominal shape was normal, the abdominal muscles were soft, and no obvious mass was touched. The liver transaminase, direct and indirect bilirubin in the blood were normal, γ Glutalmy transpeptidase (346 u/l) increased. Abdominal computed tomography (CT) revealed a circular cystic mass in the right lobe of the liver, about 41 mm × 29 mm × 53 mm in size, with clear boundary, and multiple linear partitions were seen in it. Intrahepatic bile duct dilatation was also found. On contrast enhanced CT scans, the capsule and septum enhanced, but no enhancement was found inside the tumor. The medial side of the tumor was adjacent to the main portal vein, and the portal vein ran normally, with local compression of the right branch changing (Figure 2). Subcutaneous edema was found in bilateral iliolumbar, lower abdominal wall and proximal thigh. Spleen, kidney and pancreas were normal. The mass was diagnosed as MHL.

**Figure 3:**
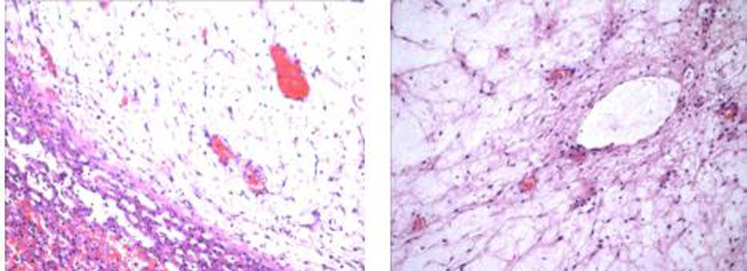
Nonmalignant proliferation of hepatocytes and fibroblasts could be seen in the edematous collagen matrix.

The operation was performed on the eighth day after birth. During the operation, it was found that the tumor originated from the fifth, sixth and seventh hepatic segment and the corresponding hepatic segment were removed. The tumor was grayish brown, measuring 70 × 55 × 40 mm. Multiple cysts with a diameter of 5–35 mm could be seen, and cysts were filled with yellow liquid. Part of the liver was gelatinous. Microscopically, nonmalignant proliferation of hepatocytes and fibroblasts could be seen in the edematous collagen matrix (Figure 3). The pathological findings were consistent with MHL.

The infant recovered and was discharged on the 6th day after operation. Follow-up at 2 years showed a thriving young girl, and there was no tumor recurrence.

### Literature review

**Figure 4:**
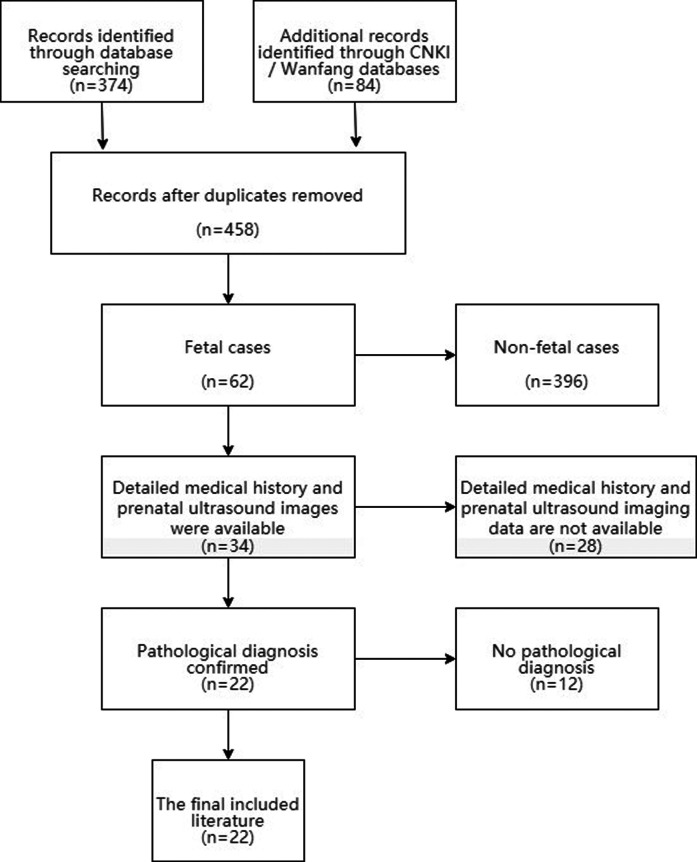
Flow chart showing the results of the literature search.

For a systematic review of the literature, PubMed, CNKI and Wanfang databases were searched to retrieve the related literature of fetal mesenchymal hamartoma of the liver from January 1980 to March 2021. We selected articles for further reading based on the title and abstract. Inclusion criteria: (1) Liver masses were found in the fetus; (2) Detailed medical history and prenatal ultrasound imaging data. (3) The pathological diagnosis was MHL. We created a PRISMA flow chart showing the results of the literature search (Figure 4).

## Results

After reviewing and complying with the inclusion criteria, 22 articles were listed (4–25). Including our patient, there are 24 reported cases of fetal MHL. There were 8 male and 16 female fetuses. Nine cases (37.5%) were found in the second trimester and 15 cases (62.5%) in the third trimester. The gestational age ranged from 15 weeks to 38 weeks, and the median gestational age was 31.5 weeks. The pregnant women were 23 to 38 years old, with a median age of 28.5 years. Two pregnant women had underlying diseases. There were 9 cases (37.5%) complicated with placental abnormalities, including 5 cases of mesenchymal stem villous hyperplasia of the placenta (MSVHP), 3 cases of placental thrombosis, and 1 case of honeycomb polycystic placenta (Table 1). The diameter of the tumor ranged from 2.0 cm to 15 cm. 20 cases were cystic, 2 cases were mixed and solid respectively. 22 patients had low or no blood flow signal, and 2 patients had high blood flow signal. After surgery or autopsy, 12 cases were found to be located in the right lobe of the liver, 7 in the left lobe, and 2 in the left and right lobes. 10 cases were intrahepatic and 14 cases were exogenous. 9 cases had polyhydramnios, 5 cases had cardiac abnormalities such as pericardial effusion or ventricular dilatation, and 8 cases had pulmonary abnormalities such as diaphragm elevation, small thorax and mild pulmonary insufficiency. Only 3 cases were correctly diagnosed as MHL before operation (Table 2). Five fetuses died *in utero*, three died within one week after birth, and the other 16 survived long term. There were 6 cases of spontaneous delivery and 1 case died in perinatal period. 13 cases of cesarean section, 2 cases died in perinatal period. Antenatal puncture and drainage were performed in 3 cases. After birth, 18 cases underwent neonatal tumor resection, and 15 cases survived, 3 cases died during the perioperative period. Among the 18 cases, 16 cases had complete tumor resection, while 2 cases had most of the tumors removed. Two cases underwent laparoscopic surgery. Three cases performed staged surgery for the tumor. Perinatal biopsy was performed, followed by radical surgery at 7 weeks, 10 weeks and 20 months postpartum. In two cases, liver cysts recurred again after operation, and finally became smaller and subsided after observation.

**Table 1:** Information about pregnant women.

First author/year	Gestational age (W)	Age of pregnant women (year)	Reproductive history	Maternal disease	Placenta
Foucar/1983	33	33	G2P0	Proteinuria, edema, hypertension, thrombocytopenia	Thick placenta, placental vasodilation with thrombosis
Hirata/1990	38	24	G3P2	–	Normal
Brian/1992	35	27	G3P2	–	Normal
BESSHO/1996	31	30	G1P0	–	Normal
Bejvan/1997	26	23	G1P0	–	–
Tovbin/1997	29	24	G4P2	–	Honeycomb polycystic placenta
Littlewood/1998	15	28	G4P2	–	–
Dickinson/1999	26	25	G4P3	–	–
Kitano/2000	34	27	G2P1	–	MSVHP
Mittermayer/2002	36	31	G2P0	–	–
Tsao/2002	19	29	–	–	Normal
Tsao/2002	33	28	–	–	Thick placenta, extensive dilation of aneurysmal branches of the placenta and chorionic veins with old and persistent thrombosis
Kamata/2003	26	–	–	–	Normal
Ramírez-Garrido/2003	37	–	–	–	Normal
LAberge/2005	19	30	G1P0	–	MSVHP
Badu/2007	32	31	G1P0	–	MSVHP
Cignini/2007	36	38	G2P1	–	Normal
Cornette/2009	33	34	G5p4	–	Normal
WAN/2009	29	25	G1P0	–	Multifocal villitis and intervillus thrombosis
Tortoledo/2010	22	28	G1P0	–	MSVHP
Kodandapani/2011	38	25	G1P0	Pregnant heart disease	Normal
Harris/2013	20	32	G2P1	–	MSVHP
Alamo/2016	22	33	G3P1	–	Normal

**Table 2:** Fetal manifestations.

First author/year	Sex	Fetal mass						Comorbidity	Prenatal diagnosis
		Mass size (cm)	Cystic or solid	Mass flow	lobe of liver	Intrahepatic or extrahepatic	Compression symptoms		
Foucar/1983	F	—	Polycystic	—	Both	Intrahepatic	Fetal growth restriction and oligohydramnios	No	Not clear
Hirata/1990	M	6 × 8	Polycystic	no flow	Left	extrahepatic	Polyhydramnios	No	Not clear
Brian/1992	M	14 × 10	solid	—	Right	extrahepatic	Bilateral hydrocele and hydramnios	No	Not clear
BESSHO/1996	M	6 × 5 × 5	Polycystic	—	Right	Intrahepatic	Polyhydramnios, pericardial effusion, systemic edema, testicular effusion	No	Not clear
Bejvan/1997	F	8 × 8 × 7	Polycystic	low flow	Right	extrahepatic	Diaphragmatic upward displacement, pericardial effusion	No	Lymphangioma
Tovbin/1997	F	3.8 × 2.7	Polycystic	no flow	Right	Intrahepatic	no	No	Not clear
Littlewood/1998	F	6.7 × 4.7 × 4.1	Cystic	—	Right	extrahepatic	no	Intestinal malrotation	Not clear
Dickinson/1999	F	8 × 7 × 6	Cystic and solid	no flow	Left	Intrahepatic	Umbilical vein dilatation, cardiac dilatation, biventricular dilatation, pericardial effusion	No	Mesenchymal hamartoma of the liver with heart failure
Kitano/2000	F	15 × 11.2 × 7.4	Polycystic	no flow	Both	Intrahepatic	no	No	Not clear
Mittermayer/2002	M	10.5 × 11 × 10	Polycystic	—	Left	extrahepatic	no	No	Not clear
Tsao/2002	M	—	Cystic	—	—	extrahepatic	no	No	Not clear
Tsao/2002	F	13 × 13 × 7	Cystic	—	—	extrahepatic	Polyhydramnios, intra amniotic hemorrhage, fetal small thorax, intraventricular hemorrhage, ventricular dilatation	No	Not clear
Kamata/2003	F	8 × 7 × 5	Polycystic	—	Left	extrahepatic	Slight elevation of left diaphragm	No	Mesenchymal hamartoma of the liver
Ramírez-Garrido/2003	M	4.9 × 4.1	Polycystic	no flow	Right	extrahepatic	Slight elevation of right diaphragm	No	Not clear
LAberge/2005	F	8.0 × 5.6 × 7.0	Polycystic	low flow	Right	Intrahepatic	Polyhydramnios, chest compression, heart tilt, bilateral ventricular wall thickening	No	Hepatic cystic hamartoma
Badu/2007	F	—	Cystic	—	Left	Intrahepatic	—	No	Not clear
Cignini/2007	F	7 × 5 × 5	Cystic and solid	low flow	—	extrahepatic	Polyhydramnios	No	Not clear
Cornette/2009	F	Maximum diameter 9.4cm	Polycystic	low flow	Right	extrahepatic	Polyhydramnios, diaphragmatic upward displacement	No	Mesenteric cystic lymphangioma
WAN/2009	F	Maximum diameter 12cm	Cystic	high flow	Right	Intrahepatic	Enlarged heart, obvious right heart	No	Hemangioma
Tortoledo/2010	F	2.4 × 2.0	Cystic	low flow	Left	extrahepatic	Mild pulmonary insufficiency	Hamartoma of lung	Lymphangioma
Kodandapani/2011	F	7 × 8	Cystic	—	Right	extrahepatic	Mild pulmonary insufficiency	No	Mesenteric cyst, ovarian cyst or repeated cyst
Harris/2013	M	9.8 × 9.1 × 10	Cystic	no flow	Right	Intrahepatic	—	No	oophoritic cyst
Alamo/2016	M	1.8 × 1.8 × 2.0	solid	low flow	Left	extrahepatic	Polyhydramnios	No	Neuroblastoma, abdominal teratoma
Liu/2019	F	4.6 × 2.9	Polycystic	low flow	Right	Intrahepatic	—	No	Not clear

## Discussion

MHL was first described by Mares in 1903 and named by Edmondson in 1956 (26). Histologically, it is composed of loose mesenchymal tissue, bile duct, connective tissue and hepatocytes, accompanied by cysts formed by mesenchymal degenerative areas or dilated bile duct and lymphatic vessels. Although MHL is the second most common benign liver tumor in childhood, fetal MHL is still a rare event. In our study, 24 cases of fetal MHL diagnosed by pathology were found by prenatal ultrasound in Chinese and English literature. Only 3 cases were correctly diagnosed before delivery, and the perinatal mortality rate was as high as 37.5%. It's necessary to summarize the previous cases and discuss the diagnosis and treatment of perinatal fetal MHL.

### Pathogenesis

The underlying pathogenesis of MHL is not clearly defined. Several hypotheses have been proposed for the etiology. Developmental anomalies, biliary obstruction, regional ischaemia, or disordered hyperplasia after liver injury have all been proposed as playing a role in its genesis (27, 28). However, the current discovery of the cytogenetics and molecular genetics showed that HML could be a real tumor. Recurrent genetic alterations identified in HML include androgenetic-biparental mosaicism (ABM) and chromosomal rearrangements which result in activation of chromosome 19q microRNA cluster (C19MC) (29). Meanwhile, sporadic HMH lesions are frequently associated with a translocation involving MALAT1 gene at chromosome 11q13 and C19MC gene at 19q13.4 (30). Appelaniz-Ruiz et al. reported two pediatric cases of mesenchymal hamartoma of the liver associated with germline DICER1 pathogenic variants (31). El Demellawy et al. described an atypical HML with a tandem triplication of chromosomal segment at chromosomem 1q44 (32). Meanwhile, rare cases of HML in the setting of Beckwith-Wiedemann syndrome (BWS) have been reported (33). HML may also be part of the expanding spectrum of findings of BWS (34). In addition, the other intriguing reports of rare concurrent infantile hemangioendothelioma and MHL (35, 36), and whether the two have a common pathogenesis is unclear. It is possible that MHL is etiologically heterogeneous. Overall, more cases are needed to further clarify the pathogenesis of HML.

### Clinical presentation

In this group, fetal MHL was found by prenatal imaging, and the pregnant women had no obvious clinical symptoms. Fetal MHL mainly occurs in the second and third trimester of pregnancy. Littlewood et al. (10) first discovered fetal MHL at the 15th week of pregnancy. MHL is considered to be a tumor associated with the growth of hilar structures during liver development. Embryological studies showed that the portal area appears at the 12th week of the fetal liver, and begins to secrete bile at the 14th week. In this group of cases, the lesions were not discovered until 15 weeks later, corresponding to the development time of fetal liver. The median age of pregnant women was 28.5 years old (23–38 years old), and all of them were younger than 35 years old, except one who was 38 years old. Twelve of them had given birth to healthy children without liver tumors. There were no obvious underlying diseases in pregnant women except case 1, who had a history of proteinuria, edema, hypertension and thrombocytopenia before pregnancy, and case 21, who had gestational heart disease。Only two pregnant women had an increase in alpha- fetoprotein and one had an abnormal increase in β-human chorionic gonadotropin (4, 11, 12).

The male and female fetuses in this group were 8 (33.3%) and 16 (66.7%) respectively, which is different from the previous studies in which the male incidence rate of infant cases was higher than that of female (37). In this study, the male perinatal mortality rate was 25% (2 cases), while the female perinatal mortality rate was as high as 43.8% (7 cases), which may also be the reason why the proportion of male cases in infancy was higher than that of female cases. The specific mechanism needs to be further studied.

### Imaging findings

Fetal MHL was mainly located in the liver or the lower margin of the liver, a small number of large exogenous masses occupied most of the abdominal cavity, and the source of the lesions could not be determined. In 6 cases, the lesions were intrahepatic, and in 11 cases, the tumors were located at the lower edge of the liver lobe, the upper end of the right kidney, and the lower end of the diaphragm. The lesion site could not be determined in 7 cases. Finally, it was confirmed by operation or autopsy that 10 cases were intrahepatic lesions, 14 cases were exogenous lesions. Among the 7 cases of tumors whose source could not be determined before delivery, 5 cases were exophytic, the diameter of tumors ranged from 7 cm to 13 cm, and some tumors occupied most of the abdominal cavity, and even extended to the pelvic cavity. Among the 7 cases, 2 cases were misdiagnosed as lymphangioma and 1 case was misdiagnosed as ovarian cyst. On the contrary, prenatal ultrasound is relatively easy to determine the source of intrahepatic lesions, but the source of mass cannot be determined in some intrahepatic lesions. Harris (25) reported a case in which the entire right lobe of the liver was occupied by a cystic mass, which was misdiagnosed as an ovarian cyst prenatal.

Ultrasonography of fetal MHL shows cystic and solid masses of varying sizes in the abdominal cavity. Most of them are cystic, including multicystic and unicystic, showing hypoechoic or anechoic, and a few are mixed or solid. Solid components can also be seen at the edge of the cystic MHL, and septa of varying thickness can be seen in the mass. Color Doppler Flow Imaging shows that most of the tumors have low blood flow or no blood flow signal, and a few have high blood flow signal. In this group, 20 cases (83.3%) were cystic, and only 2 cases (8.33%) were mixed and solid respectively. Some studies believe that MHL is solid at first. With the growth of the mass, interstitial cystic change is accompanied by secondary fluid accumulation, lymphatic obstruction and expansion in the cyst, which gradually expands the cystic area in the mass, and occupies most or even all of the mass, making the mass appear cystic solid or mainly cystic (37). Through the studies of ultrasonic and pathological on children's solid MHL, Wang Xiaoman et al. (38) believed that spongy MHL could not be identified by ultrasound due to its tiny pores, and thus showed solid changes, but its pathological findings still had numerous small cysts, still in line with the pathological characteristics of polycystic. In this group, 23 cases (92.9%) had low or no blood flow signal, and only 1 case (7.1%) had high blood flow signal. Cases with high blood flow signal are easy to be misdiagnosed. Cases with high blood flow signal are prone to misdiagnosis. WAN (21) misdiagnosed a HML with high blood flow signal as hemangioma. Because fetal ultrasound showed a vascular cystic mass in the liver, postpartum ultrasound and MRI also suggested high blood flow signal.

Although MHL is a benign tumor, fetal MHL can grow rapidly, resulting in displacement of surrounding organs or compression symptoms. In this group, 9 cases had polyhydramnios, 5 cases had cardiac abnormalities such as pericardial effusion or ventricular dilatation, and 8 cases had pulmonary abnormalities such as diaphragm elevation, small thorax and mild pulmonary insufficiency. MHL can compress the inferior vena cava and umbilical vein, which can lead to congestive heart failure, including fetal heart enlargement, cardiac dilatation, pericardial effusion, fetal edema, etc. Fluid accumulation in cysts and decreased albumin production due to compression of the liver itself further increase the risk of edema. Compression from the mass causes obstruction of the upper digestive tract, leading to polyhydramnios. In addition, the fetus is at risk of lung hypoplasia due to the compression of the diaphragm by the mass.

Fetal magnetic resonance imaging (MRI) may help to clarify the source of the lesion and the nature of the tumor. Compared with ultrasound, MRI has higher resolution in soft tissue examination. The visual field is larger, and can be scanned in any section to display the full picture of the fetus. MRI is less affected by the mother's condition, not by the fetal bone and amniotic fluid volume, and less affected by the operator's technical level. Currently, MRI has been increasingly used in the diagnosis of fetal diseases, and has become an important supplement to obstetric ultrasound, and can provide additional information that cannot be detected by ultrasound. In this group, a total of 5 fetuses received prenatal MRI examination, mainly showing high signal in T2-weighted images and low signal in T1-weighted images, suggesting cystic components. Hepatic hamartoma was diagnosed by MRI in 2 of 5 cases (40%), while prenatal ultrasound accurately diagnosed only 1 case. In another 2 cases, the tumor was derived from liver by judging the accurate relationship between the tumor and surrounding organs, and only 1 case of huge cystic mass was misdiagnosed as ovarian cyst. Therefore, MRI further examination is recommended when prenatal abdominal masses cannot be clearly diagnosed.

Concommitant placental abnormalities have been reported in 9 cases (37.5%) of HML, including 5 cases with mesenchymal stem villous hyperplasia of the placenta (MSVHP), 3 cases with placental thrombosis and 1 case with honeycomb polycystic placenta. MSVHP is a recently proposed placental disease, which is characterized by diffuse edema of stem villi, relatively normal terminal villi, and often accompanied by abnormal villi vasodilatation. The increased incidence of HML with PMD and the morphological similarities of the changes seen in both the placenta and liver, suggests a possible common developmental mechanism. The association between HML and placental mesenchymal dysplasia was initially described by Alwaidh et al. (39). Both entities share androgenetic-biparental mosaicism as an underlying mechanism; paternal uniparental disomy results in imbalanced expression of imprinted loci in androgenetic cells which may lead to abnormal tissue phenotypes (40).

### Treatment of fetal MHL

The main cause of death in fetuses and neonates with mesenchymal hamartoma was progressive abdominal distension owing to a rapidly expanding, fluid-filled, cystic hepatic mass that produced severe respiratory distress and compression of normal, intraabdominal blood vessels and other structures (41). The primary task of treatment is to ensure the stability of fetuses and neonates vital signs.

In this group, 3 patients underwent biopsy and drainage of mass before delivery. Tsao et al. (14) described a large fetal intraperitoneal unilocular HML found at 19 weeks, which began to decrease after three cyst aspirations. A healthy boy was born at 35 weeks and the cyst was removed laparoscopically. Bejvan (9) reported an invasive puncture at 26 weeks for a rapidly growing polycystic mass with pericardial effusion. Due to the rapid re-accumulation of fluid after puncture, the authors re-inserted two pigtail catheters, one between the largest cyst and the other between the cyst and the amniotic cavity, but the results were not satisfactory. Premature rupture of membranes occurred at 30 weeks and the baby was delivered by cesarean section. Postnatal percutaneous drainage also had no significant effect. On the 21st day after birth, laparotomy was performed to confirm and remove a pedicled hepatic mesenchymal hamartoma. Kamata et al. (15) found a polycystic mass in the left upper abdomen at 26 weeks, and had an emergency caesarean section at 30 + 5 weeks due to maternal hypertension. The fetus underwent percutaneous cyst puncture and drainage before delivery, and bleeding was found at the puncture site after delivery, with hemoglobin as low as 5.7 g/dl. Emergency laparotomy was forced, and hemorrhage within the tumor was finally found. Thus, in polycystic MHL, drainage may not achieve the desired effect of tumor reduction due to unobstructed and incompatible communication between different cysts. Since the liquid will be quickly regenerated, the drainage is repeated frequently. And prenatal treatment is only temporary decompression, which can't reduce the need for postpartum surgical resection. Meanwhile, puncture may lead to fetal bleeding. In addition, invasive fetal treatment carries a risk of premature rupture of membranes and premature delivery. It is also worth noting that previous studies have shown that the cytology of cyst aspirates cannot be used to determine the diagnosis (38), so this invasive procedure has no role in aiding diagnosis. Therefore, from these only three puncture cases, it can be inferred that prenatal cyst puncture may be of therapeutic significance for monocystic MHL, but not for polycystic MHL, and may cause harm to the fetus.

Early cesarean section can improve the fetal survival rate. In this group, 13 cases underwent early cesarean section, and finally 3 cases died after delivery. The other 11 cases underwent observation or natural delivery. Finally, 5 cases died *in utero* and 1 case died after delivery. The rapid growth of fetal MHL volume can often produce compression symptoms. MHL compresses the inferior vena cava and umbilical vein, which can lead to congestive heart failure, resulting in fetal edema. There were 5 cases of fetal heart enlargement, dilatation of the heart cavity, pericardial effusion and fetal edema, among which 4 cases died in perinatal period. 8 cases suffered from chest compression, such as diaphragmatic muscle elevation, thoracic diminution and pulmonary insufficiency, of which 4 cases died in perinatal period. Cardiopulmonary compression symptoms caused by MHL may be an important cause of fetal death. Cesarean section is beneficial to fetal survival before serious cardiopulmonary dysfunction occurs. In addition, vaginal delivery is reported to carry a risk of cyst rupture and death, as well as a risk of soft tissue dystocia, so cesarean section should be the preferred method of delivery.

Since MHL has often caused compression symptoms of peripheral organs, and the tumor volume still grows rapidly, timely and effective treatment is needed. Some new genetic evidences suggest the MHL may be neoplastic *ab initio*. In addition, Clinical and histological evidence has strongly suggested that UES can develop within a pre-existing HML. Malignant transformation may occur several years after an incomplete resection of the lesions (42). Therefore, the preferred treatment is complete resection of the mass. Open resection or laparoscopic surgery can be performed according to the size and anatomical location of the mass. When one-stage resection is difficult, staged operation still has good effect. Regular radiological follow-up should be carried out after operation, and local recurrence may subside. But it should be noted that it is important to sample different parts of the HML to exclude areas of malignant transformation (2). For some neonates with unstable vital signs, tumor vascular interventional therapy can be considered (43). Liver transplantation is the most effective treatment for unresectable symptomatic HML (33, 44).

In conclusion, MHL is a rare benign tumor of fetal liver, which is often found in the second and third trimester of pregnancy, and there is no obvious predisposing population. The typical ultrasonographic findings of MHL are cystic and solid masses in the liver region, which are mainly cystic and rarely solid, and most of them are exophytic growth. The Color Doppler Flow Imaging shows that most of the tumors have low blood flow or no blood flow signal, and a few have high blood flow signal. MHL is often accompanied by compression symptoms such as cardiac enlargement, dilatation of cardiac cavity, pericardial effusion, fetal edema, elevation of diaphragm, diminution of thorax, pulmonary insufficiency, and increased amniotic fluid. MHL is often associated with placental mesenchymal dysplasia. Prenatal cyst puncture may have therapeutic significance for single cystic MHL, but not for polycystic MHL, and may cause damage to the fetus. Cesarean section may improve the survival rate of newborns. Complete resection of tumor after birth is an effective treatment. When one-stage operation is difficult to perform, vascular intervention or partial resection is feasible, and then re-staged operation still has a good prognosis. Liver transplantation is the most effective treatment for unresectable symptomatic HML.

## Data Availability

The original contributions presented in the study are included in the article/Supplementary Material, further inquiries can be directed to the corresponding author/s.
